# Anterior Pituitary Hormones in Blood and Cerebrospinal Fluid of Patients in Neurocritical Care

**DOI:** 10.17925/EE.2022.18.1.71

**Published:** 2022-06-13

**Authors:** Henriette Beyer, Nicole Lange, Armin H Podtschaske, Jan Martin, Lucia Albers, Alexander von Werder, Jürgen Ruland, Gerhard Schneider, Bernhard Meyer, Simone M Kagerbauer, Jens Gempt

**Affiliations:** 1. Department of Neurosurgery, Technical University Munich, School of Medicine, Klinikum rechts der Isar, Munich, Germany; 2. Department of Anesthesiology, Technical University Munich, School of Medicine, Klinikum rechts der Isar, Munich, Germany; 3. Department of Medical Informatics, Statistics and Epidemiology, Technical University Munich, School of Medicine, Klinikum rechts der Isar Munich, Germany; 4. Department of Neuroendocrinology, Technical University Munich, School of Medicine, Klinikum rechts der Isar, Munich, Germany; 5. Department of Clinical Chemistry, Technical University Munich, School of Medicine, Klinikum rechts der Isar, Munich, Germany

**Keywords:** Anterior pituitary hormones, cerebrospinal fluid, circadian rhythm, neurocritical care

## Abstract

Background: Anterior pituitary hormones in blood follow a circadian rhythm, which may be influenced by various factors such as intracranial pathologies. In cerebrospinal fluid (CSF), pituitary hormones have been collected only selectively and circadian rhythm has not yet been investigated. This pilot study analysed diurnal variations of anterior pituitary hormones in patients in neurocritical care to determine whether circadian rhythmicity exists in these patients. Possible influences of intracranial pathologies were also investigated. Blood and CSF concentrations were assessed simultaneously to explore the value of blood concentrations as a surrogate parameter for CSF levels. Methods: Blood and CSF samples of 20 non-sedated patients were collected at 06:00, noon, 18:00 and midnight, and analysed for adrenocorticotropic hormone (ACTH), cortisol, thyroid-stimulating hormone (TSH) and insulin-like growth factor-1 (IGF-1) concentrations at each of the four time points. ACTH and IGF-1 were measured by sandwich chemiluminescence immunoassay. Cortisol and TSH were measured by electrochemiluminescence immunoassay. Results: Results showed inconsistent circadian rhythms. Less than 50% of the patients showed a circadian rhythmicity of ACTH, cortisol, TSH or IGF-1. Significance of diurnal variations was only present for blood concentrations of TSH. Correlations between blood and CSF concentrations were strong for cortisol and TSH. Conclusions: CSF concentrations were only in the measurable range in some of the patients. No clear circadian rhythmicity could be identified, except for TSH in blood. Absence of significant diurnal variations could be explained by the underlying pathologies or disturbing influences of the intensive care unit. Blood concentrations of cortisol and TSH may be suitable surrogate parameters for CSF.

In physiological conditions, the pituitary gland contributes to proper body functions and homeostasis. The assumed circadian rhythm of anterior pituitary hormones seems to be an important part of the hormonal balance. Cortisol release triggered by adrenocorticotropic hormone (ACTH) forms the main part of the body's ‘stress response’, which is crucial for a patient's reaction to critical events such as trauma or operations. The circadian rhythm of serum cortisol and ACTH is described in the established literature with minima at midnight and maxima in the early morning.^1^ Thyroid-stimulating hormone (TSH), which shows maximum blood levels at midnight and minimum levels at noon, interacts with the corticotroph axis and participates, amongst other things, in heat regulation and myocardial function.^1^ Similarly, insulin-like growth factor-1 (IGF-1) contributes to metabolism and the immune system. A more complex rhythm with several maxima and minima, is expected for the somatotroph axis, especially for growth hormone (GH), whereas IGF-1 levels in blood showed maxima in the morning and minima in the early evening.^2^ Disturbance of pituitary hormone homeostasis can have far-reaching consequences such as influencing heart rate, blood pressure and glucose homeostasis, and account for muscle wasting and memory impairment.^1^

Circadian rhythmicity can be disrupted by elements such as brain injury, medication or unnatural environments including those with noise and artificial light, which are common in a modern intensive care unit (ICU), and these disruptors have been discussed extensively in the literature.^3,4^ As many body functions are modulated by diurnal variations of plasma glucocorticoid levels, it might be possible that disturbances of circadian rhythmicity lead to hormonal imbalances that essentially influence clinical outcome.

Central nervous system (CNS) studies on pituitary hormones and peptides mostly concern sex steroids and the hypothalamic neuropeptides oxytocin and arginine-vasopressin. These substances seem to exert cognitive and behavioural as well as neuroprotective effects.^5,6^

As the brain is not directly accessible and cerebrospinal fluid (CSF) collection involves invasive procedures, little is known about the occurrence and metabolism of anterior pituitary hormones in the CNS of humans. Only a few predominantly older studies on this topic exist^7,8^ and they are mostly confined to daily samples; studies performing serial measurements are scarce.^9^

The aim of this study was to investigate circadian rhythmicity of pituitary hormones of awake patients with intracranial pathologies in the absence of sedatives. We wished to determine: 1) whether anterior pituitary hormones are detectable in CSF using common methods; 2) whether these CSF concentrations reflect blood concentrations; and 3) whether there is also a circadian rhythmicity in the CNS, as described for blood concentrations of certain hormones such as cortisol.

## Methods

The study cohort included non-sedated, awake patients with external ventricular drainage. Only patients who were orientated and able to give written informed consent were included in the study. Exclusion criteria were underage patients, application of catecholamines or sedatives, CSF infections (defined as fever, pathological glucose or lactate concentrations in CSF compared with blood), cortisol substitution and refusal to participate in the study. Patients underwent follow-up 3 months after disease onset. All but three patients had good neurological outcome and were largely independent in everyday life. Thus, we were able to recruit a relatively homogeneous cohort for the study. The study was approved by the ethics committee of the Technical University of Munich, School of Medicine (reference number 5459/12).

Blood and CSF were collected from a pre-existing arterial line and external ventricular drainage. Collection was scheduled at 06:00, noon, 18:00 and midnight. Each sample was analysed for cortisol, ACTH, TSH and IGF-1 levels, amounting to a total of eight data points per patient per point in time.

The samples were analysed by the institutional Department of Clinical Chemistry. For blood concentrations, the laboratory reference range was taken as the benchmark for normal values. For CSF concentrations, no reference ranges exist in the routine diagnostics of our hospital.

Both CSF and blood levels of ACTH and IGF-1 were measured by sandwich chemiluminescence immunoassay using a Liaison^®^ XL analyser (DiaSorin, Saluggia, Italy; reference number 313221 for ACTH and 313231 for IGF-1).

Cortisol and TSH levels in blood and CSF were determined by electrochemiluminescence immunoassay using a cobas e411^®^ analyser (Roche Diagnostics, Mannheim, Germany; reference number 06 687733 190) for cortisol and a cobas 8000^®^ analyser (Roche Diagnostics; reference number 11 731459 122) for TSH.

Hormone concentrations in blood and CSF were first analysed at an individual level. The analysis determined the detectability of the hormones with common analytical methods and then, for blood concentrations only, the position within or outside the reference range. The temporal course in the individual patients was graphically displayed and visually checked for the presence of a possible circadian rhythm. In a second step, cumulative analysis of the entire patient cohort was carried out.

Circadian rhythm was defined as minima and maxima at the time points described in the established literature. This means that cortisol and ACTH show maxima in the early morning and minima at midnight; TSH would have its maximum at midnight and minimum around noon.^1^ For IGF-1, maxima in the early morning and minima in the early evening are described.^2,3^

Statistical analysis was performed using IBM SPSS Statistics^®^ version 25 (IBM Corp., Armonk, NY, USA) and R^®^ version 3.5.2 and 3.1.0 (the R foundation for Statistical Computing, Vienna, Austria). The significance of diurnal variations of hormone levels was determined by Friedman test and, if appropriate, verified by Wilcoxon and Bonferroni test as *post hoc* analyses. Correlations between blood and CSF levels were determined using Spearman's rank correlation coefficients. A p value of <0.05 was considered statistically significant.

For statistical calculations, concentrations below the assays' detection limit were handled as being equal to the threshold.

## Results

A total of 20 patients were included in the study (12 female, 8 male; mean age 54.4 years [range 34–75]). Regarding diagnoses, 15 patients suffered aneurysmatic subarachnoid haemorrhage (Hunt and Hess II–IV), three patients showed hydrocephalus malresorptivus after resection of intracranial tumours, and two patients suffered traumatic subarachnoid haemorrhage.

**Table 1:** Number of measurements below the detection limit

	Blood				CSF			
	ACTH	Cortisol	TSH	IGF-1	ACTH	Cortisol	TSH	IGF-1
Total number of measurements	79	80	80	80	79	80	80	80
Below detection limit	12	1	0	0	67	0	0	57

*Due to technical reasons, ACTH in blood and CSF was missing once in one patient.*

*ACTH = adrenocorticotropic hormone; CSF = cerebrospinal fluid; IGF -1 = insulin-like growth factor-1; TSH = thyroid-stimulating hormone.*

**Figure 1:**
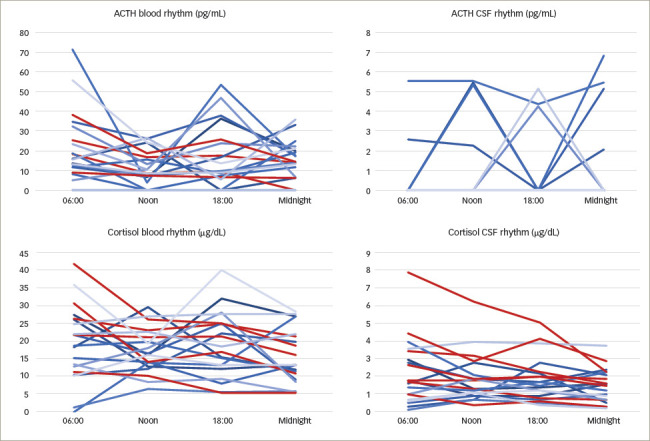
Individual time courses of adrenocorticotropic hormone and cortisol concentrations in blood and cerebrospinal fluid

*Time courses that follow a circadian rhythm described in the literature are shown in red.*

*ACTH = adrenocorticotropic hormone; CSF = cerebrospinal fluid.*

### Circadian rhythmicity of blood and cerebrospinal fluid hormone levels

In most cases, hormone concentrations in the blood could be determined. In the CSF, ACTH and IGF-1 were below the detection limit in the vast majority of cases (*Table 1*). CSF cortisol and TSH concentrations corresponded to values described in the literature, whereas ACTH and IGF-1 concentrations were lower in our study.^10–13^

#### Analysis at individual patient level

**Figure 2:**
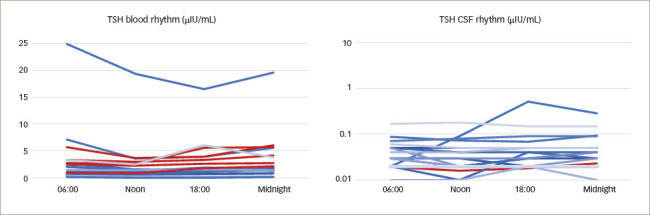
Individual time courses of thyroid-stimulating hormone concentrations in blood and cerebrospinal fluid

**Figure 3:**
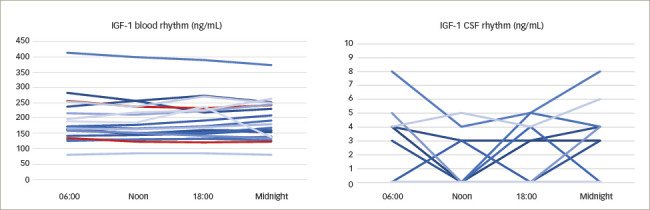
Individual time courses of insulin-like growth factor-1 concentrations in blood and cerebrospinal fluid

Individual time courses of blood and CSF concentrations of ACTH, cortisol, TSH and IGF-1 are shown in *Figures 1–3*. Patient curves that correspond to rhythms described in the literature are coloured red.

Adrenocorticotropic hormone: ACTH blood levels were detectable at each time point in 15 patients. Concentrations ranged from 5.3 pg/mL to 71.9 pg/mL, with a median of 15.75 pg/mL. ACTH blood levels within the laboratory reference range (4.7–48.8 pg/mL) were found in 12 patients. One sample at 18:00 was missing due to an intervention outside the ward.

Four patients showed physiological rhythm in blood as described in established literature, which is comparable to the assumed rhythm of ACTH (*Figure 1*).^1^

Only one patient showed ACTH levels in CSF above the threshold of determination at all sampling time points. CSF concentrations ranged from 4.4 pg/mL to 5.6 pg/mL. A total of 13 patients consistently had no measurable ACTH levels in CSF. No circadian rhythm could be shown (*Figure 1*).

Cortisol: Cortisol levels above the detection limit could be assessed consistently in blood samples of 19 patients. Concentrations ranged from 1.2 μg/dL to 42.4 μg/dL, with a median of 17.2 μg/dL. Five out of 20 patients showed physiological cortisol levels for all samples (reference range 10–25 μg/dL).

*Time courses that follow a circadian rhythm described in the literature are shown in red.*

*CSF = cerebrospinal fluid; TSH = thyroid-stimulating hormone.*

*Time courses that follow a circadian rhythm described in the literature are shown in red CSF = cerebrospinal fluid; IGF -1 = insulin-like growth factor-1.*

Six patients experienced the assumed circadian rhythm in blood, with maxima in the early morning and minima around midnight (*Figure 1*).^1^

CSF cortisol levels were measurable for all patients. Concentrations ranged from 0.1 μg/dL to 8.0 μg/dL, with a median of 1.58 μg/dL. Seven patients experienced a circadian rhythm of cortisol in CSF similar to that assumed in blood (*Figure 1*).

Thyroid-stimulating hormone: TSH blood levels were detectable in all samples and ranged from 0.07 μlU/mL to 24.94 μlU/mL, with a median of 1.67 μlU/mL. A total of 13 patients showed TSH levels within the laboratory reference range (0.27–4.20 μlU/mL) at each time point.

Five patients experienced the assumed circadian rhythm in blood, with maxima at midnight and minima around noon (*Figure 2*).

TSH CSF concentrations were entirely above the threshold of determination. Concentrations ranged from 0.01 μlU/mL to 0.52 μlU/ mL in CSF, with median of 0.03 μlU/mL. One patient showed a circadian rhythm similar to that assumed in blood (*Figure 2*).^1^

Insulin-like growth factor-1: IGF-1 was detectable in all blood samples, 12 of which showed concentrations within the reference range (age dependent: 25–39 years 100–250 ng/mL; 40–54 years 90–245 ng/mL; >55 years 54–205 ng/mL). No patient showed reduced IGF-1 concentrations compared with the normal range. IGF-1 in blood ranged from 80 ng/mL to 413 ng/mL, with a median of 171.5 ng/mL.

Two patients showed a circadian rhythm of IGF-1 in blood, with maxima in the early morning and minima in the early evening^1^ (*Figure 3*).

Three patients showed measurable IGF-1 levels in CSF consistently. For 11/20 patients, IGF-1 levels could not be determined in any CSF sample. Concentrations ranged from 3 to 8 ng/mL, with a median of 4 ng/mL. One patient showed a value of 153 ng/mL in CSF. Considering the other IGF-1 concentrations in CSF, we viewed this value as an outlier value. No circadian rhythm was apparent (*Figure 3*).

#### Analysis at the cohort level

Regarding the whole patient cohort, pituitary hormones in blood showed significant diurnal variations only for TSH (p=0.003). *Post hoc* analysis showed significant variations between 06:00 and noon, as well as between noon and midnight.

**Figure 4:**
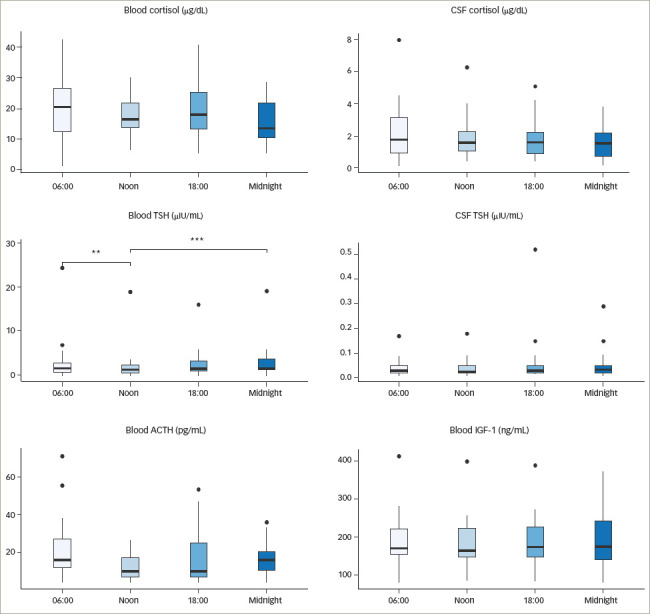
Hormone concentrations at the different time points

** : p<0.05.

***: p<0.01.

*All available values were included in the analysis. Only TSH blood concentrations at 06:00 and noon, as well as at noon and midnight differed significantly.*

*ACTH = adrenocorticotropic hormone; CSF = cerebrospinal fluid; IGF -1 = insulin-like growth factor-1; TSH = thyroid-stimulating hormone.*

CSF ACTH and IGF-1 levels were below the detection limit in more than 50% of the collected samples, and therefore we did not perform an analysis for diurnal fluctuations in the whole patient cohort in these cases.

*Figure 4* shows a summarized representation of the blood and CSF concentrations of all available measurements at the individual sampling times in the form of boxplots. Medians and interquartile ranges are depicted in *Table 2*.

### Correlations of blood and cerebrospinal fluid hormone levels

**Figure 5:**
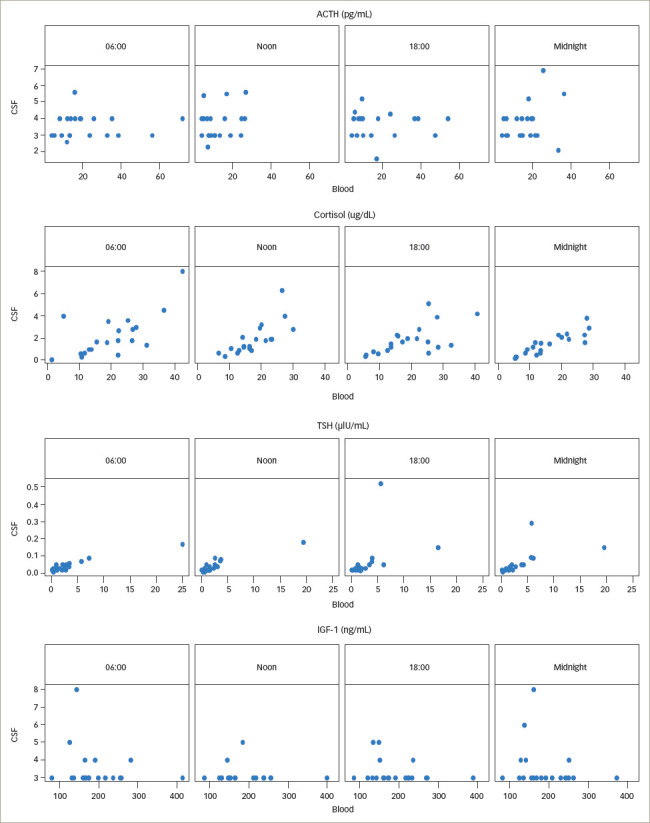
Scatterplots depicting correlations between blood and cerebrospinal fluid levels of adrenocorticotropic hormone, cortisol, thyroid-stimulating hormone and insulin-like growth factor-1

Correlations between blood and CSF concentrations varied depending on hormone and time point. Four independent data sets, each consisting of 20 data points, could be constructed from the sample data. No significant correlations were found between blood and CSF values of ACTH or IGF-1 at the different time points. Cortisol and TSH exhibited a significant positive correlation for all points in time (0.61<r<0.89) (*Figure 5*). The strongest correlation between blood and CSF values was found in midnight samples (r=0.89, p<0.05). All correlation coefficients are shown in *Table 3*.

## Discussion

In our study of non-sedated, awake patients in neurocritical care, only a minority showed the assumed circadian rhythms in blood as described in the literature. In the CSF, no significant diurnal fluctuations of cortisol, ACTH, TSH or IGF-1 could be detected when regarding the whole patient cohort. Only cortisol and TSH showed strong correlations of blood and CSF levels, and therefore, only in these cases could blood concentrations act as surrogate parameter for CSF concentrations.

**Table 2:** Hormone concentrations in cerebrospinal fluid and blood

	Time of sample				p
	06:00	Noon	18:00	Midnight
Hormone concentrations, median (IQR)					
Blood ACTH, pg/mL	16 (12–28)	10 (7–17)	10 (7–25)	16 (10–20)	0.090
Blood cortisol, μg/dL	21 (13–27)	16 (14–22)	18 (13–25)	13 (11–22)	0.057
Blood TSH, μlU/mL	1.74 (0.81–2.92)	1.45 (0.68–2.55)	1.67 (1.15–3.5)	1.66 (1.44–3.91)	0.003
Blood IGF-1, ng/mL	170 (155–221)	164 (146–223)	173 (147–227)	174 (140–242)	0.300
CSF cortisol, μg/dL	1.75 (0.92–3.12)	1.55 (1.05–2.28)	1.60 (0.87–2.22)	1.54 (0.7–2.15)	0.075
CSF TSH, μlU/mL	0.03 (0.02–0.05)	0.03 (0.02–0.05)	0.03 (0.02–0.05)	0.04 (0.02–0.05)	0.122

*Only TSH blood concentrations showed significant variations between different time points.*

*ACTH = adrenocorticotropic hormone; CSF = cerebrospinal fluid; IGF -1 = insulin-like growth factor-1; IQR = interquartile range; TSH = thyroid-stimulating hormone.*

Our data indicate a disturbed circadian rhythm of the hypothalamic– pituitary–adrenal axis hormones in blood as assumed by the established literature. A plausible explanation for this disruption is stress caused by severe illness or disturbing environmental influences in an ICU setting.^14,15^ Noise and artificial light, as well as disturbed sleep–wake cycles, are potential stressors and cause disturbance of circadian rhythmicity.^3,4,16^

In our findings, blood cortisol levels were increased in 11 out of 20 patients, indicating stressful stimuli. The blood cortisol circadian rhythm as described in the established literature was only present in 30% of the patients.

TSH showed the most robust rhythm in blood of all four investigated hormones: 9 out of 20 (45%) patients had their maxima at midnight or 06:00, with minima at noon or 18:00.

Furthermore, only 20% of the patients in our study showed a circadian rhythm of ACTH in blood. For IGF-1 blood levels, there were even fewer patients (10%) showing circadian rhythmicity in the blood.

Therefore, our results are similar to the findings of Schneider et al. who showed that impairment of pituitary hormones after traumatic brain injury and aneurysmal subarachnoid haemorrhage has a sequence: ACTH is mainly affected, TSH was influenced least.^17^

In general, brain injury due to trauma, surgery or ischaemia may cause hypothalamic–pituitary–adrenal axis disturbances, with subsequent impaired hormone secretion and disturbed circadian rhythm.^18^ Disturbances of circadian rhythm in patients in the ICU have been described previously but, in contrast to our study, focused on analgosedated patients.^19^

Above all, the definition of circadian rhythmicity is questionable. As conclusions on the physiological circadian rhythm cannot directly be drawn from our patients, a comparison with similarly designed studies is reasonable. Of particular interest is the study by Zetterling et al., because of the analogous study design.^14^ The authors investigated 20 patients, sampling blood for cortisol and ACTH at 06:00, noon, 18:00 and midnight. Results were categorized into normal, reversed and unspecific diurnal pattern. However, the authors' definition of a physiological circadian rhythm differs from the description in the current article. Zetterling et al. defined ‘normal’ as maximum cortisol levels at 06:00 or noon, and ‘inversed’ as minimum at 06:00 or noon.^14^ If this definition is applied to the current study, 13 out of 20 patients (65%) would exhibit a ‘normal diurnal pattern’. Thus, a change of definition would lead to markedly changed findings regarding the prevalence of a circadian rhythm for these data.

Fundamental research of rhythmicity of TSH and the somatotroph axis was performed by Patel et al. in 1972.^20^ Their definition of circadian rhythm of TSH was less strict than for the corticotroph axis and is still the current reference for a circadian rhythm of TSH. The authors' findings within a group of six subjects showed maxima at between 02:00 and 04:00, and minima between 18:00 and 20:00, results that were partially confirmed by the results of the current study.

IGF-1 was preferred over GH in our study because the rhythmicity of GH has previously been described as very complex. The role of IGF-1 in intracranial pathologies is relatively well studied, and endogenous production in the CNS has been described;^2,21^ however, diurnal variations of IGF-1 concentrations have, to the best of our knowledge, not been investigated to date.

Our understanding of the circadian rhythm of the somatotroph axis is based on work by Winer and Shaw 1990.^2^ The authors included 12 patients and described a pulsatile secretion pattern. In contrast, some laboratory datasheets point out that IGF-1 measurement is possible throughout the whole day because of lack of circadian pattern, proving diverging views of circadian behaviour of IGF-1.^22^ In the current study, as far as a circadian rhythmicity of IGF-1 is concerned, 90% of the patients showed no significant diurnal variations.

As CSF sampling always requires invasive procedures, little is known about pituitary hormones in the CSF and their possible circadian fluctuations. Blood and CSF concentrations differ: pituitary hormones in CSF are only present at a fractional amount of serum levels.^23^ Data on ACTH in CSF are scarce and have included patients with various diseases; however, findings suggest that CSF ACTH levels are slightly higher than plasma levels and that the blood–brain barrier is impermeable to ACTH.^8,24^ Circadian rhythmicity with a peak in the evening could be shown in primates.^25^ The obtainable measurements in our study suggest no rhythm in CSF for ACTH. Cortisol showed the expected rhythm in CSF in only seven out of 20 patients. Our findings did do not suggest any kind of rhythm of TSH in CSF. For IGF-1, a full set of measurements was only obtained for three patients. The other measurements were again below the detection limit. Therefore, circadian rhythm of IGF-1 in CSF can be neither confirmed nor refuted based on these data.

Hormone concentrations in CSF may depend not only on the molecular size but also on blood levels.^26^ Therefore, the permeability of the blood– brain barrier, as well as the blood–CSF barrier, could also influence CSF concentrations of pituitary hormones. This permeability may be also affected by stress and, therefore, stressful conditions may alter CSF concentrations. However, this fact is discussed controversially in the literature.^27^ In patients with traumatic brain injury, strong correlations of cortisol and progesterone my indicate impaired blood–brain barrier or disturbed hormone metabolism within the brain.^28^

*No significant correlations were found between blood and CSF values of ACTH or IGF-1 at the different time points. Cortisol and TSH exhibited a significant positive correlation for all points in time.*

*ACTH = adrenocorticotropic hormone; CSF = cerebrospinal fluid; IGF -1 = insulin-like growth factor-1; TSH = thyroid-stimulating hormone.*

**Table 3:** Spearman's rank correlation coefficients for correlations between blood and cerebrospinal fluid levels

		06:00	Noon		18:00		Midnight	
r	p		r	p	r	p	r	p
ACTH	0.206	0.384	0.168	0.478	-0.098	0.689	0.168	0.479
Cortisol	0.621	**0.003**	0.812	**<0.0001**	0.611	**0.004**	0.894	**<0.0001**
TSH	0.755	**0.0001**	0.801	**<0.0001**	0.65	**0.002**	0.878	**<0.0001**
IGF-1	-0.083	0.729	0.137	0.566	-0.1	0.674	-0.231	0.327

*p<0.05 was considered statistically significant, as shown in bold*

*ACTH = adrenocorticotropic hormone; CSF = cerebrospinal fluid; IGF -1 = insulin-like growth factor-1; TSH = thyroid-stimulating hormone.*

Correlation of serum and CSF levels of pituitary hormones is discussed controversially, with several authors either confirming^29^ or refuting^23,24^ a correlation. In the current study, the statistical evaluations showed different results for each hormone. For cortisol and TSH, the moderate-to-strong positive correlation allows us to draw inferences about CSF levels from blood sampling. This would allow the investigation of larger patient cohorts in different contexts without the need for external CSF drainage. ACTH and IGF-1 showed only weak correlations between blood and CSF. However, for these hormones, analysis was hindered by the fact that many values were below the detection limit. This suggests that electrochemiluminescence immunoassay cannot be recommended for determination of ACTH and IGF-1 CSF concentrations as this method does not seem to be sensitive enough. However, we chose these analytical methods because they can be easily applied within clinical routine in a hospital's laboratory. For detection of CSF ACTH and IGF-1 levels, more sophisticated methods are needed, but cortisol and TSH can be detected in the CSF by the common routine assays used in our study.

Little is known about pituitary hormones and their metabolism in the CNS. For example, sex hormones are supposed to be synthesized within the CNS and to exert neuroprotective functions.^5^ Upregulated expression of IGF-1 within the brain is described as a response to post-traumatic brain damage.^21^ Impaired cerebral hormone metabolism could be a cause of poor outcome in acute cerebral disease. Strong positive correlations of blood and CSF concentrations may indicate impaired integrity of the blood–brain barrier. Alternatively, a lack of correlation may hint to endogenous hormone synthesis and independent metabolism within the brain, as discussed for sex steroids, oxytocin and arginine-vasopressin.^5,6^ Differing diurnal secretion patterns in blood and CSF may also suggest independent peripheral and central hormone metabolism. As circadian rhythmicity was absent in a large percentage of patients in our study, this question cannot be answered satisfactorily.

Our study was designed to contribute to a better understanding of pathophysiological processes in neurocritical care patients, but the number of included patients was too small to evaluate practical implications. However, it is conceivable that the integrity of the blood– brain barrier and the circadian rhythm may be worth monitoring to improve patients' internal rhythm with targeted application of hormones. This could lead to re-establishment of a patient's physiological condition. Furthermore, improved monitoring may provide more information about prognosis and recovery processes. Until now, routine hormone measurement in blood, and especially in CSF, has been too expensive and time consuming. The results of this pilot study indicate that it is worth investigating these conditions in more detail in further studies.

### Limitations

Several limitations to our study are evident. First, due to the invasive nature of CSF sampling, only a small sample size could be generated.

Second, blood and CSF samples taken at the four time points were put on ice and immediately transported to the laboratory for further processing. A control group of healthy individuals would therefore have had to be hospitalized for the observation period of 24 hours. Unfortunately, this was not possible for organizational and logistical reasons, so that a control group had to be omitted in this study design, even if only blood samples had been taken from it.

Third, patients included in the study showed intracranial pathologies that might contribute to hypothalamic–pituitary disturbances. To mitigate this factor, only non-sedated patients undergoing normal (in the context of ICU) sleep–wake cycles were included, as we assumed their circadian rhythms were more likely to be intact. Thus, we achieved a relatively homogeneous study cohort. Most of the patients had a good neurological outcome, so that a correlation analysis between hormone concentrations and neurological outcome would not have yielded a satisfactory result. Further studies with more severely ill patients are necessary to investigate associations between hormone concentrations and neurological status.

Furthermore, electrochemiluminescence immunoassay/sandwich chemiluminescence immunoassay methods, as commonly used in routine diagnostics, are often not sensitive enough for CSF hormone levels. Studies employ heterogeneous methods to determine CSF hormone levels and a standard method is yet to evolve.^30,31^

## Conclusions

A circadian rhythm for hormones in blood was only present in a minority of patients. Either the rhythm is disturbed by brain injury or by other factors originating from the ICU.

Significant diurnal variations of hormones in blood at the cohort level were only shown for TSH. For other hormones, no definitive statement can be made as CSF concentrations in particular were often below the detection limit. Disturbed hormone metabolism and disruption of the blood–brain and blood–CSF barriers may be a possible influencing factor.

CSF and blood levels for TSH and cortisol were positively correlated. Therefore, blood concentrations of these hormones could act as an indicator for CSF concentrations, which would facilitate further studies.
